# Angucyclinones rescue PhLOPS_A_ antibiotic activity by inhibiting Cfr-dependent antibiotic resistance

**DOI:** 10.1128/mbio.01791-23

**Published:** 2023-11-28

**Authors:** Adam J. Schaenzer, Annia Rodriguez Hernandez, Kaitlyn Tsai, Christian Hobson, Danica Galonić Fujimori, Gerard D. Wright

**Affiliations:** 1 Michael G. DeGroote Institute for Infectious Disease Research, McMaster University, Hamilton, Ontario, Canada; 2 Department of Biochemistry and Biomedical Sciences, McMaster University, Hamilton, Ontario, Canada; 3 Department of Cellular and Molecular Pharmacology, University of California San Francisco, San Francisco, California, USA; 4 Chemistry and Chemical Biology Graduate Program, University of California San Francisco, San Francisco, California, USA; 5 Quantitative Biosciences Institute, University of California San Francisco, San Francisco, California, USA; 6 Department of Pharmaceutical Chemistry, University of California San Francisco, San Francisco, California, USA; MedImmune, Gaithersburg, Maryland, USA

**Keywords:** adjuvants, natural anti-microbial products, antibiotic resistance, methyltransferase, Cfr, angucyclinones

## Abstract

Methylation of the ribosome is a prevalent strategy conferring resistance to antibiotics that inhibit protein synthesis. Methylation is catalyzed by several distinct enzymes, many encoded on mobile genetic elements widespread in bacteria. One enzyme that is increasingly common in Gram-positive pathogens is Cfr. This radical S-adenosyl-L-methionine rRNA methylase confers resistance to the antibiotics phenicols, lincosamides, oxazolidinones, pleuromutilins, and streptogramin A (PhLOPS_A_) via methylation of A2503 of the 23S rRNA of the large ribosomal subunit. One strategy to safeguard the utility of these antibiotics is to identify inhibitors of Cfr-dependent methylation that may rescue antibiotic activity. We screened our in-house library of microbial natural product extracts against Cfr^+^*Escherichia coli* and identified an extract from *Streptomyces* WAC01849 that potentiated the PhLOPS_A_ antibiotic linezolid in a Cfr-dependent manner. We isolated the active angucyclinones 8-*O*-methyltetrangomycin (MTN) and 8-*O*-methyltetrangulol (MTL) from this extract. Both compounds selectively potentiated representatives of the PhLOPS_A_ antibiotics in *Escherichia coli* and methicillin-resistant *Staphylococcus aureus* in a Cfr-dependent manner. Finally, we observed inhibition of Cfr-mediated ribosome methylation in cells treated with MTN and MTL. Our findings suggest that MTN and MTL interfere with Cfr-dependent methylation to protect the efficacy of the PhLOPS_A_ antibiotics and offer a unique chemical scaffold that may be applied in developing clinically useful Cfr inhibitors.

**IMPORTANCE:**

Cfr is an antibiotic resistance enzyme that inhibits five clinically important antibiotic classes, is genetically mobile, and has a minimal fitness cost, making Cfr a serious threat to antibiotic efficacy. The significance of our work is in discovering molecules that inhibit Cfr-dependent methylation of the ribosome, thus protecting the efficacy of the PhLOPS_A_ antibiotics. These molecules are the first reported inhibitors of Cfr-mediated ribosome methylation and, as such, will guide the further discovery of chemical scaffolds against Cfr-mediated antibiotic resistance. Our work acts as a foundation for further development of molecules that safeguard the PhLOPS_A_ antibiotics from Cfr.

## INTRODUCTION

The bacterial ribosome has proven to be an exquisite antibiotic target; to date, eight clinically relevant classes of antibiotic drugs target multiple sites across both ribosomal subunits (1). Two classes, the aminoglycosides and tetracyclines, target the small ribosomal subunit, while six (the macrolides, streptogramins, pleuromutilins, lincosamides, oxazolidinones, and phenicols) target the large subunit. Through various mechanisms, these antibiotics block the successful translation of mRNA into functional proteins.

As with all other antibiotics, resistance to ribosome-targeting antibiotics is a growing threat to modern healthcare. Modification of the ribosome by methylation of rRNA is a particularly effective antibiotic resistance strategy, often conferring resistance to multiple classes of antibiotics (2). Most rRNA methyltransferases catalyze the transfer of a “CH_3_^+^” equivalent from a methyl donor, commonly S-adenosyl-L-methionine (SAM), to a nucleophilic nitrogen of the base or the C2 hydroxyl group of the ribose moiety of an rRNA nucleoside via an S_N_2 mechanism releasing S-adenosylhomocysteine (SAH) as a product (3, 4). The introduced methyl group is small enough to minimally disrupt ribosome structure and function but serves to sterically exclude the antibiotic from its binding site. Since many ribosome-targeting antibiotics possess partially overlapping binding sites, methylation of a strategic locus in the rRNA can efficiently render multiple antibiotic classes ineffective.

One rRNA methylating enzyme of growing concern is Cfr. Unlike typical methyltransferases, Cfr is a radical SAM methylating enzyme that primarily methylates the electron-deficient *sp*^2^-hybridized carbon at position 8 (C8) of adenosine A2503 of the 23SrRNA (*Escherichia coli* numbering) within the peptidyl transferase center (5). Cfr also uses SAM as a methylating agent, but the reaction requires two molecules of SAM in a two-step process. The first step involves typical SAM-mediated S_N_2 methylation of an active site Cys releasing SAH. In the second step, a second SAM is homolytically cleaved to generate methionine and a 5′-dA radical, which abstracts a hydrogen from the active site *S*-methyl Cys, releasing adenosine and generating a highly reactive methylene radical that transfers to the target carbon of A2503 (6, 7).

While C-2 is also targeted by the housekeeping radical SAM methylating enzyme RlmN and has minimal effects on antibiotic resistance, methylation of C8 confers resistance to the phenicols, lincosamides, oxazolidinones, pleuromutilins, and streptogramins A (known as the “PhLOPS_A_” phenotype) (8). The *cfr* gene family is readily mobilized and has minimal fitness cost (9), allowing for dissemination among various Gram-positive (and select Gram-negative) pathogens (10). In one clinical methicillin-resistant *Staphylococcus aureus* (MRSA) isolate, Cfr was found alongside the methyltransferase ErmB, which modifies the 23S rRNA at positions A2058/A2059, a combination that effectively blocks all clinically relevant antibiotic classes that target the large ribosomal subunit (11). Consequently, Cfr poses a serious and growing threat to the utility of many antibiotics.

One strategy to safeguard current antibiotics is the use of antibiotic adjuvants. Antibiotic adjuvants augment antibiotics while lacking significant antibiotic activity themselves (12). The classic example of an antibiotic adjuvant is a small molecule inhibitor of an antibiotic resistance enzyme; co-administration of the antibiotic with such an inhibitor protects the former from resistance, prolonging the life span of antibiotic therapy. The clinical success of beta-lactam/beta-lactamase inhibitor combinations (13, 14) has encouraged the search for antibiotic adjuvants that target other antibiotic resistance enzymes such as Cfr. Here we present a screen of our in-house natural product extract library that revealed 8-*O*-methyltetrangomycin (MTN) and 8-*O*-methyltetrangulol (MTL) as inhibitors of Cfr-mediated antibiotic resistance.

## RESULTS

### High-throughput screen identifies potentiators of the PhLOPS_A_ antibiotic linezolid

**Fig 1:**
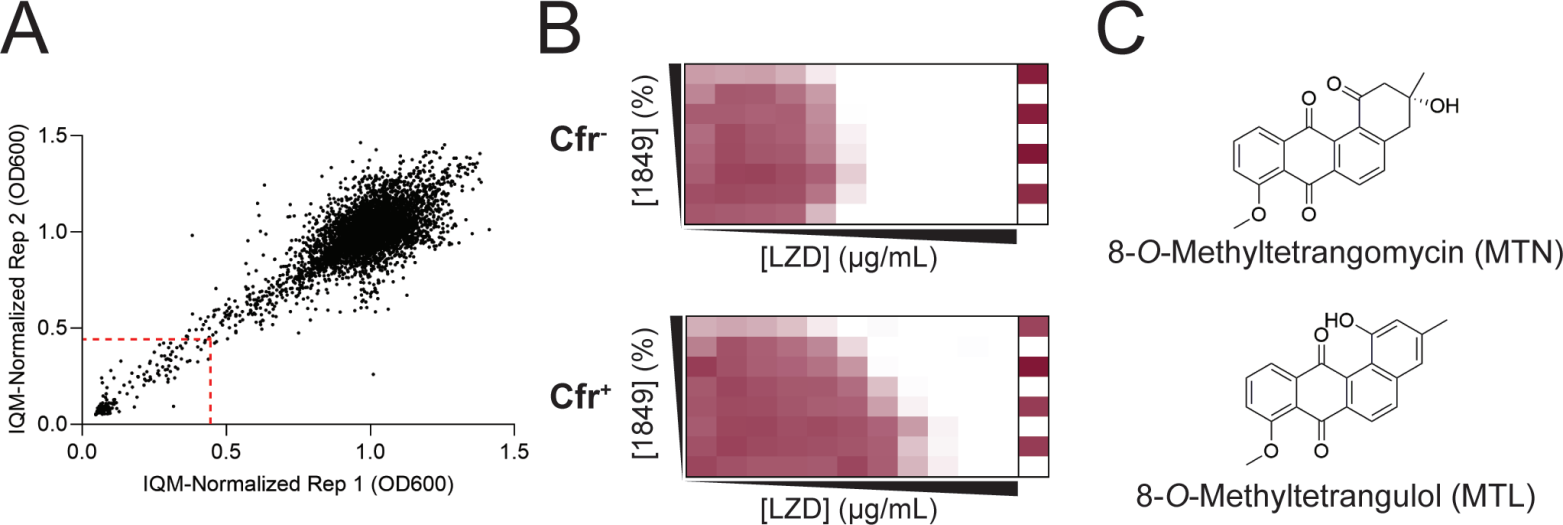
A microbial natural product extract screen identifies 8-*O*-methyltetrangomycin (MTN) and 8-*O*-methyltetrangulol (MTL) as potentiators of linezolid (LZD) in the presence of Cfr. (**A**) Replica plot of the interquartile mean (IQM)-normalized replicates of natural product library. Red dashed line denotes 3σ hit cutoff. (**B**) Synergy grid assay of *E. coli* BW25113 Δ*bamB*Δ*tolC* ± Cfr. Bacterial growth is denoted by maroon color. Max (1849) extract = 1%; max (LZD) = 128 µg/mL. (**C**) Structures of MTL.

To identify potential Cfr inhibitors, we screened our in-house natural product extract library for extracts that could rescue the PhLOPS_A_ antibiotic activity of linezolid in a Cfr-expressing background. The screen was performed in duplicate against *E. coli* BW25113 Δ*bamB*Δ*tolC* (15) carrying pGDP2:*cfr* (*E. coli*-Cfr) supplemented with 1/16th of the minimal inhibitory concentration (MIC) of linezolid. We chose the *E. coli* BW25113 Δ*bamB*Δ*tolC* background as it is efflux deficient and highly permeable to compounds that generally are excluded from the *E. coli* outer membrane (15). Data points were normalized to the interquartile mean (IQM) of their respective replicate (16), and the IQMs of each trial were averaged to get a library IQM average of 0.969. Our hit threshold was set to 3σ below the IQM average (0.443), resulting in 248 hits (Fig. 1A). After secondary screens for reproducibility and linezolid dependence, we followed up on the extract from *Streptomyces* WAC01849 as it gave the most robust phenotype that was reproducible and linezolid dependent.

We reasoned that if a component of the WAC01849 extract is a Cfr inhibitor, bacterial growth inhibition in the presence of linezolid should depend on the presence of Cfr. To test this hypothesis, we performed synergy checkerboard grid experiments with crude WAC01849 extract and linezolid against *E. coli*-Cfr and its empty vector control (*cfr*^−^). Indeed, we observed potentiation of linezolid with increasing concentrations of the extract against our *cfr*^+^ strain only, consistent with the presence of a Cfr inhibitor in the extract (Fig. 1B).

We next performed activity-guided purification to identify the active component of the WAC01849 extract, resulting in the purification of two compounds: a yellow solid and a cinnamon-brown powder. The proton nuclear magnetic resonance (NMR) spectrum for the yellow solid matched that of MTN (17), while the proton NMR spectrum for the cinnamon-brown powder matched that of MTL (17) (Fig. 1C). Both compounds are type II polyketides belonging to the angucyclinone family of compounds. The angucyclinones, and their glycosylated derivatives, the angucyclines, possess a benz[a]anthraquinone core modified by a diverse suite of tailoring enzymes (18). While they are diverse in their ornamentation, many angucyclinones share a common biosynthetic intermediate, the core benz[a]anthraquinone UWM6, created by a conserved cassette of six genes: beta-ketoacyl synthase, chain length factor, acyl carrier protein, short-chain dehydrogenase/reductase, and a pair of cyclases (18). A scan of the *Streptomyces* WAC01849 genome using anti-SMASH identified a single biosynthetic gene cluster (BGC) containing the UWM6 biosynthetic cassette alongside genes for tailoring enzymes with high homology to those of the kiamycin BGC (Fig. S1).

### MTL and MTN specifically potentiate PhLOPS_A_ antibiotics

**Fig 2:**
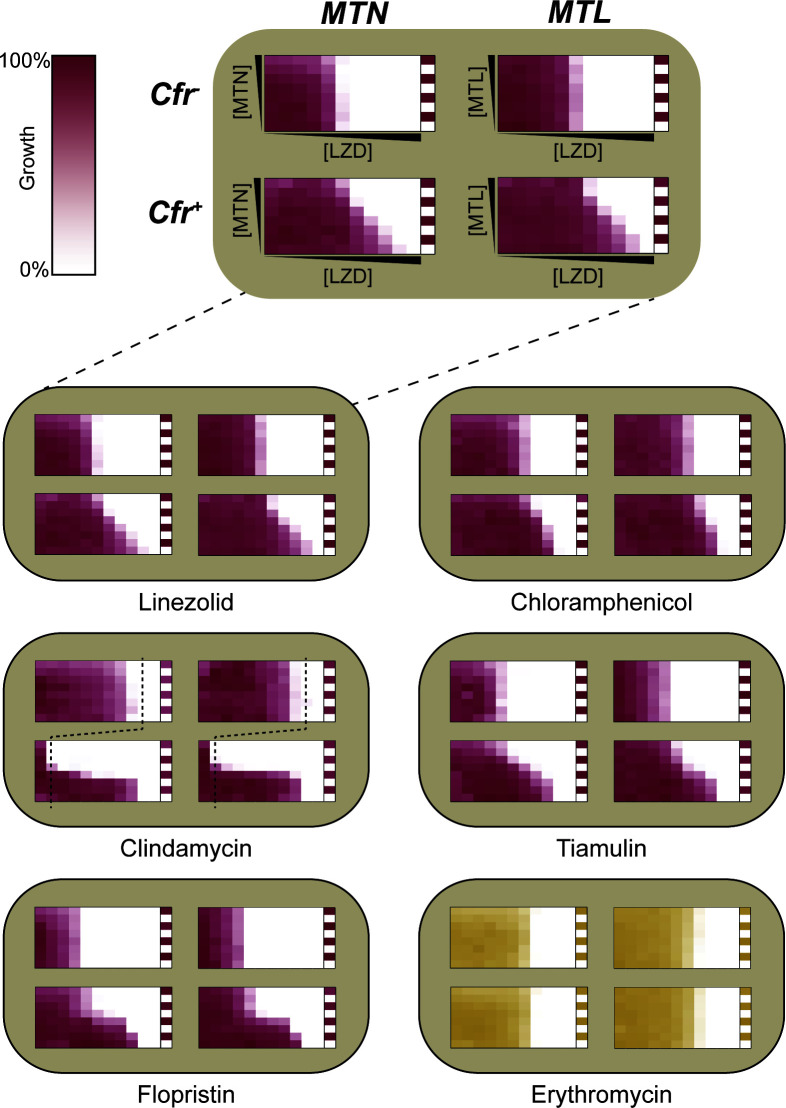
MTN and MTL selectively potentiate PhLOPS_A_ antibiotics against *E. coli* in a Cfr-dependent manner. For all synergy grids, max MTN and MTL are 30 and 3 µM, respectively. Synergy grids are grouped by the PhLOPS_A_ antibiotic on their respective *x*-axes. Within groups, synergy grids are as follows (starting top-left, clockwise): Cfr^−^, MTN; Cfr^−^, MTL; Cfr^+^, MTL; Cfr^+^, MTN. Max (linezolid) = 128 µg/mL, max (chloramphenicol) = 8 µg/mL, max (tiamulin) =8 µg/mL, max (flopristin) = 8 µg/mL, max (erythromycin) = 4 µg/mL, max (clindamycin) = 4 µg/mL (Cfr^−^) and 1024 µg/mL (Cfr^+^). For clindamycin, vertical dashed lines denote MIC for Cfr^−^ strain. All synergy grids are representative of three biological replicates.

MTN and MTL were discovered as suppressors of Cfr-mediated linezolid resistance. If these compounds are general inhibitors of Cfr, they should potentiate other PhLOPS_A_ antibiotics in a *cfr*-dependent manner. Therefore, we tested other representatives of the PhLOPS_A_ antibiotics in our synergy grid assay. Both MTN and MTL could potentiate each PhLOPS_A_ representative (Fig. 2; Table S1); however, MTL was approximately 10-fold more potent than MTN. Intriguingly, the lincosamide clindamycin and type A streptogramin flopristin were most dramatically affected, their MICs dropping rapidly as the concentration of potentiator increased. The oxazolidinone linezolid and the pleuromutilin tiamulin displayed a more gradual decrease in MIC, while chloramphenicol was the least affected of the PhLOPS_A_ antibiotics. Notably, both MTN and MTL failed to potentiate any antibiotic in the absence of the *cfr* gene, consistent with Cfr dependence. Neither compound could potentiate erythromycin, a 14-membered macrolide that targets the nascent peptide exit tunnel of the large ribosomal subunit (Fig. 2), nor ampicillin, a cell wall-targeting antibiotic (Fig. S2). Our data demonstrate that MTN and MTL specifically potentiate the PhLOPS_A_ antibiotics in a Cfr-dependent manner.

### MTN and MTL inhibit methylation of 23S rRNA residue A2503

**Fig 3:**
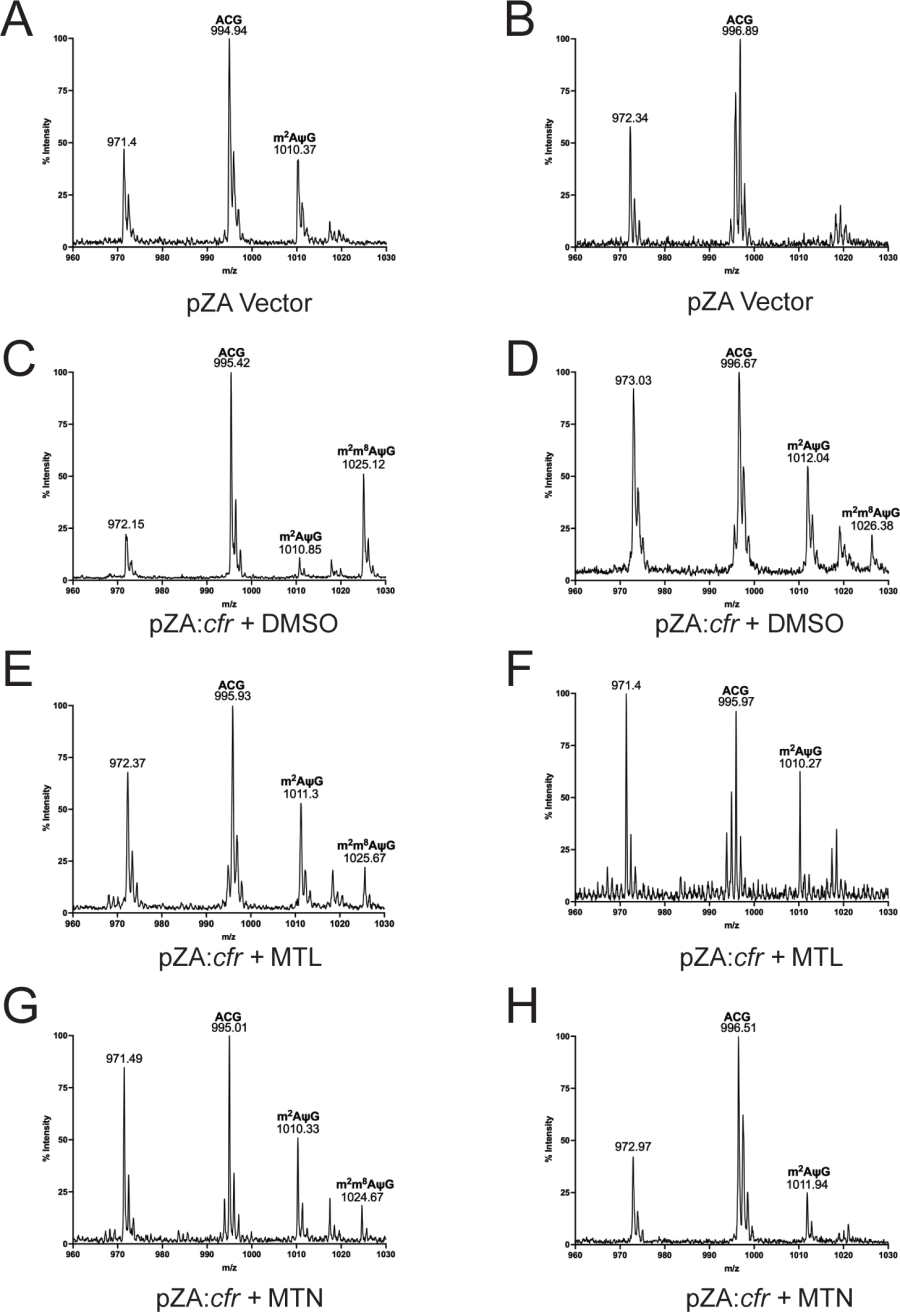
MTN and MTL inhibit methylation of A2503. (**A**) dimethyl sulfoxide (DMSO)-treated *E. coli* BW25113 Δ*bamB*Δ*tolC* pZA empty vector. (**B**) DMSO-treated *E. coli* Δ*bamB*Δ*tolC rlmN::kan* empty vector. (**C**) DMSO-treated *E. coli* BW25113 Δ*bamB*Δ*tolC* pZA:*cfr*. (**D**) DMSO-treated *E. coli* Δ*bamB*Δ*tolC rlmN::kan* pZA:*cfr*. (**E**) MTL-treated *E. coli* BW25113 Δ*bamB*Δ*tolC* pZA:*cfr*. (**F**) MTL-treated *E. coli* Δ*bamB*Δ*tolC rlmN::kan* pZA:*cfr*. (G) MTN-treated *E. coli* BW25113 Δ*bamB*Δ*tolC* pZA:*cfr*. (**H**) MTN-treated *E. coli* Δ*bamB*Δ*tolC rlmN::kan* pZA:*cfr*. MTN = 30 µM, MTL = 3 µM. A2503 from the 23S rRNA denoted by AψG species.

Given the dependence on Cfr for the potentiation phenotype, we hypothesized that the presence of MTN and MTL would impact Cfr-dependent methylation of the 23S rRNA. To test this hypothesis, we incubated *E. coli* BW25113 Δ*bamB*Δ*tolC* pZA:*cfr* in the presence or absence of MTN and MTL, purified the total RNA, and isolated the C2480-C2520 fragment of the 23S rRNA containing base A2503, the site of Cfr-mediated methylation. Following nuclease digestion, modification of RNA fragments was analyzed by matrix-assisted laser desorption ionization (MALDI) mass spectrometry (19). In the absence of *cfr* and the presence of the housekeeping methylase *rlmN*, the expected presence of adenine modified at position 2 (m^2^AψC) is observed (Fig. 3A). *In vitro* studies have shown that A2503 can be methylated by Cfr at both positions C8 and C2 with a strong preference for the former (20); however, A2503 is also methylated at the C2 position by the endogenous RlmN (21). To test the effect of identified adjuvants of Cfr-catalyzed methylation alone, we generated the corresponding rlmN knockout strain, *E. coli* BW25113 Δ*bamB*Δ*tolC rlmN::kan*. As expected, we observed the complete absence of A2503 methylation in this strain (Fig. 3B). We observed the appearance of dimethylated A2503 in rRNA fragments purified from the *cfr* and *rlmN* expressing strain (m^2^m^8^AψC, Fig. 3C), validating the predicted activity of Cfr. Introduction of pZA:*cfr* to the *rlmN* mutant restored both monomethylation (mAψC) and dimethylation (m^2^m^8^AψC), consistent with Cfr’s known ability to methylate both positions on A2503 (Fig. 3D).

Treatment with MTN or MTL led to a decrease in the intensity of the dimethylated m^2^m^8^AψC signal with a concomitant increase in the monomethyl peak (Fig. 3E and G), consistent with inhibition of Cfr-catalyzed methylation. In the rlmN null background, treatment with MTN or MTL results in a complete absence of the m^2^m^8^AψC dimethylation signal and a decrease in the mAψC monomethylation signal (Fig. 3F and H). Incubation with either MTN or MTL alone did not lead to a significant change in methylation of A2503 in the corresponding rRNA fragments isolated from *E. coli* BW25113 Δ*bamB*Δ*tolC* pZA:*Empty* (Fig. S3). These observations suggest that the two molecules identified in our screen do not significantly perturb RlmN-catalyzed methylation and support the hypothesis that MTN and MTL potentiate the PhLOPS_A_ antibiotics by inhibiting Cfr-mediated methylation of 23S rRNA.

Based on the decrease in Cfr-mediated methylation, we hypothesized that MTN and MTL directly inhibit Cfr. However, *in vitro* assays with purified Cfr demonstrated only a modest inhibition of enzyme activity (30% inhibition by MTN), suggesting a more complex or indirect mechanism of activity (Fig. S4).

### MTN and MTL potentiate PhLOPS_A_ antibiotics in MRSA

**Fig 4:**
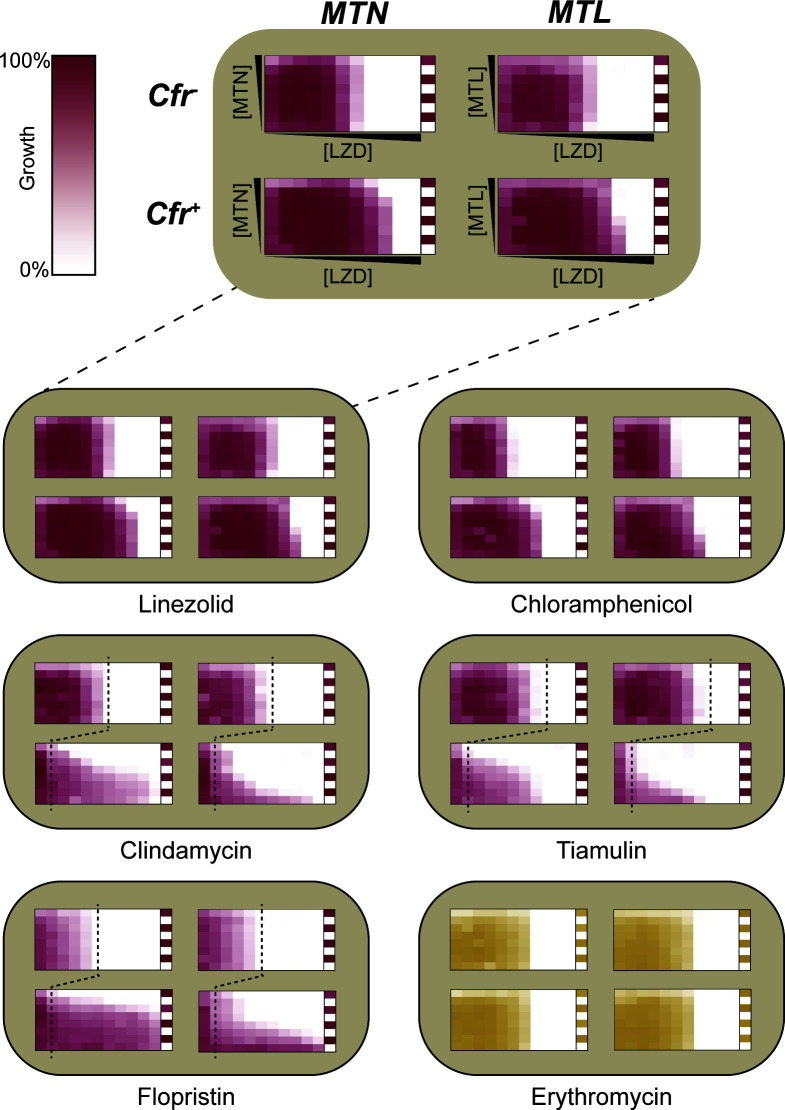
MTN and MTL selectively potentiate PhLOPS_A_ antibiotics against *S. aureus* in a Cfr-dependent manner. For all synergy grids, max (MTN) and (MTL) are 16 µM. Synergy grids are grouped and arranged as in Fig. 2. Max (linezolid) = 16 µg/mL, max (chloramphenicol) = 128 µg/mL, max (tiamulin) = 2 µg/mL (Cfr^−^) and 256 µg/mL (Cfr^+^), max (flopristin) = 8 µg/mL (Cfr^−^) and 128 µg/mL (Cfr^+^), max (erythromycin) = 1 µg/mL, max (clindamycin) = 1 µg/mL (Cfr^−^) and 32 µg/mL (Cfr^+^). For clindamycin, tiamulin, and flopristin, vertical dashed lines denote MIC for Cfr^−^ strain. All synergy grids are representative of three biological replicates.

PhLOPS_A_ antibiotics are clinically essential drugs used against various Gram-positive pathogens, particularly as alternative treatments for MRSA and vancomycin-resistant enterococci (VREs). As such, Cfr-mediated antibiotic resistance further limits therapeutic options for these pathogens. To determine if MTL and MTN could potentiate PhLOPS_A_ antibiotics against a Cfr-positive pathogen, we cloned the *cfr* gene into the MRSA strain COL (22) using the pKK30 vector with expression controlled by the constitutive *sarA* P1 promoter (COL-Cfr). COL-Cfr showed a PhLOPS_A_ antibiotic resistance profile similar to our *E. coli* strain described above, with only modest increases in linezolid and chloramphenicol MICs and a more dramatic rise in MICs for the remaining antibiotics. MTN and MTL potentiated PhLOPS_A_ against COL-Cfr, though MTN was once again less potent than MTL (Fig. 4; Table S2). Importantly, both MTN and MTL lowered the MICs of linezolid and clindamycin to their clinical breakpoints (Table 1). Both compounds exhibited rescue concentrations (defined as the minimum concentration of adjuvant required to lower the MIC to or below an antibiotic’s clinical breakpoint) in the low micromolar range. Neither compound potentiated the non-PhLOPS_A_ antibiotics erythromycin (Fig. 4) nor vancomycin (Fig. S2), demonstrating that MTN and MTL selectively potentiated PhLOPS_A_ antibiotics against *S. aureus* in a Cfr-dependent manner.

**TABLE 1:** Median rescue concentrations of adjuvants against *S. aureus* COL pKK30:*cfrA*

Adjuvant	Median rescue concentration (µM) (range)
Linezolid^*a*^	
MTN	8 (88–16)
MTL	2 (1–2)
Clindamycin^*b*^	
MTN	16 (8–16)
MTL	2 (1–2)

^*a*^ Linezolid European Committee on Antimicrobial Susceptibility Testing (EUCAST) breakpoint: susceptible at ≤4 mg/L.

^*b*^ Clindamycin EUCAST breakpoint: susceptible at ≤0.25 mg/L.

## DISCUSSION

The ribosome is a high-value target for antibiotic drugs; however, various mechanisms of antibiotic resistance jeopardize the continued utility of current ribosome-targeting antibiotics. Modifying the ribosome through rRNA methylation is of particular concern as many of the families of ribosome-targeting antibiotics possess overlapping binding sites, allowing for resistance to multiple antibiotic classes with the methylation of a single residue. Inhibiting the offending methylating enzymes could prolong the utility of currently established antibiotics. Here we present our findings of the first, to our knowledge, inhibitors of ribosome methylation by the antibiotic resistance enzyme Cfr.

Cfr confers resistance to five classes of antibiotics yielding the PhLOPS_A_ phenotype. Notably, the lincosamide clindamycin and the oxazolidinone linezolid are alternate treatments for MRSA (23), and the latter is considered a first-line therapy for VRE (24); the established presence of Cfr in clinical isolates limits treatment options for these recalcitrant pathogens. Efforts to circumvent the activity of Cfr have led to the approval of the second-generation oxazolidinone tedizolid (25) and ongoing research into the novel compound iboxamycin (26). While both compounds retain activity against Cfr^+^ pathogens, an inhibitor of Cfr itself would also be beneficial to safeguard five antibiotic classes simultaneously.

MTN and MTL are angucyclinones with a characteristic benz[a]anthraquinone core. Given the well-established biosynthesis of this benz[a]anthraquinone core, we were able to identify their likely BGC in the *Streptomyces* WAC01849 genome. The cluster contains 80% of the genes from the known kiamycin BGC with only four genes absent (Fig. S1). MTN has been proposed to be an intermediate of kiamycin biosynthesis and has been copurified with kiamycins in previous studies (27). MTN and its nonmethylated derivative, tetrangomycin, are proposed to convert non-enzymatically to MTL and tetrangulol under non-neutral conditions (27, 28). MTN and MTL may not be the final products of the WAC01849 BGC but are intermediates of kiamycin or a kiamycin-like compound. Further work with the BGC is needed to validate this hypothesis. Nevertheless, it is a reminder that an intermediate and not the final product of a BGC can have desirable bioactivity and the value of phenotype-based screens of natural product extracts.

Our MALDI experiments reveal decreased methylation of 23S rRNA A2503 in bacteria treated with our angucyclinones, consistent with decreased Cfr activity. However, preliminary experiments with MTN suggest a more complex mechanism than direct enzyme inhibition. Given Cfr’s radical SAM mechanism of action (20) and MTN’s known free radical-scavenging activity (29), it is tempting to speculate that MTN and, by extension, MTL may interfere with the reduction of iron-sulfur cluster or formation and stability of radical intermediates formed during Cfr-mediated methylation of A2503. Alternatively, if an oxygen-dependent mechanism such as redox cycling is involved, the anaerobic environment required for the *in vitro* enzyme assay may impede the inhibition phenotype. Further study is needed to elucidate the exact mechanism of action of these compounds.

Like antibiotics, antibiotic adjuvants impose a selective pressure on their targets; therefore, the evolution of resistance to adjuvants like MTN and MTL is inevitable. Future studies are needed to determine frequency of resistance and the potential mechanisms of resistance (and any fitness costs associated with them).

Angucyclinones and their glycosylated derivatives, angucyclines, possess a wide range of bioactivities, including anti-oxidant, anti-bacterial, and anti-cancer activities. Both MTN and MTL have reported anti-bacterial activity (30). However, potentiation of the PhLOPS_A_ antibiotics occurs 10-fold to 40-fold below the reported MICs and is Cfr dependent. Therefore, it is evident that MTN and MTL possess potentiation activity independent of their anti-bacterial properties.

Besides anti-bacterial activity, MTN and MTL also possess anti-cancer activity (27, 30), an undesirable trait for an antibiotic adjuvant. Such cytotoxicity must be minimized before moving forward with a lead compound. Fortunately, the benz[a]anthraquinone scaffold is readily amenable to derivatization, as evidenced by the diverse ornamentation and rearrangements of angucyclines and angucyclinones in nature (18). Cytotoxicity could be minimized via a medicinal chemistry strategy consisting of a combination of semisynthesis and synthetic biology.

This discovery and characterization of the first small molecule inhibitors of Cfr-mediated ribosome methylation augur well for downstream studies to expand our understanding of the molecular mechanism of this series and encouragement for the discovery of additional natural product chemical scaffolds with similar anti-Cfr activity. Further exploration of the angucyclinones will undoubtedly lead to insights regarding the optimal pharmacophore required to inhibit ribosome methylation by Cfr. Such information is crucial to safeguard the PhLOPS_A_ antibiotics against Cfr and prolong their clinical utility as life-saving medicines.

## MATERIALS AND METHODS

### Strains, growth conditions, and reagents

*E. coli* strains were routinely grown on Luria Broth (LB) agar at 37°C or in LB broth at 37°C shaken at 250 rpm, supplemented with 50-µg/mL kanamycin or 100-µg/mL ampicillin when required. *S. aureus* strains were routinely grown on tryptic soy agar at 37°C or in tryptic soy broth at 37°C shaken at 250 rpm, supplemented with 10-µg/mL trimethoprim when required. Strain WAC01849 was routinely grown on Bennett’s agar [10-g/L potato starch, 2-g/L casamino acids, 1.8-g/L yeast extract, 2-mL/L Czapek mineral solution (100-g/L KCl, 100-g/L MgSO_4_·7H_2_O, 120-g/L NaNO_3_, 2-g/L FeSO_4_·7H_2_O, and 2-mL/L concentrated HCl), and 15-g/L agar; pH-adjusted to 6.8] at 30°C or in *Streptomyces* antibiotic production media (SAM) (15-g/L glucose, 15-g/L soya peptone, 5-g/L NaCl, 1-g/L yeast extract, 1-g/L CaCO_3_, and 2.5-mL/L glycerol; pH-adjusted to 6.8) at 30°C, 250 rpm, except where noted. For a complete list of strains used in this study, see Table S3.

All primers and gBlock gene fragments were synthesized by Integrated DNA Technologies, Inc. For a complete list of primers used in this study, see Table S4.

### Natural product library screen

*E. coli* BW25113 Δ*bamB* Δ*tolC + pGDP2:cfrA* was grown overnight at 37°C, 250 rpm in cation-adjusted Mueller-Hinton (CAMH) broth containing 8-µg/mL linezolid. Grown culture was diluted to an OD_600_ of 0.2 in CAMH; this dilution was further diluted 1:200 in CAMH containing 16-µg/mL linezolid (DMSO = 0.0625%). Twenty-four microliters of this mixture was added by a Beckman Biomek FX^P^ liquid handler (Beckman Coulter, Inc.) to 384-well plates containing 25-µL CAMH and 1 µL of our in-house arrayed natural product library (in DMSO). Plates were incubated in an Infors HT Multitron Pro (Infors AG) at 37°C, 700 rpm, 90% humidity for 18 hours. After incubation, OD_600_ was measured on a Synergy H1 microplate reader (BioTek Instruments). The screen was performed in duplicate.

Before screening, a *Z*′ experiment was performed as above, replacing extracts with an equivalent amount of DMSO. *E. coli* BW25113 Δ*bamB* Δ*tolC + pGDP2:cfrA* was used as the negative control, and *E. coli* BW25113 Δ*bamB* Δ*tolC + pGDP2* vector was used as the positive control. The positive and negative conditions gave interquartile mean OD_600_ values of 0.057 (± 0.005) and 0.581 (± 0.062), respectively. *Z*′ was calculated as follows:


$$Z^{\prime }=1-\left(\frac{3\sigma_{+c}+3\sigma_{-c}}{\left|\mu_{-c}-\mu_{+c}\right|}\right),$$

where σ_+c_ and µ_+c_ are the standard deviation and interquartile mean of the positive condition, respectively, and σ_−c_ and µ_−c_ are the standard deviation and interquartile mean of the negative condition, respectively. The *Z*′ score was 0.62.

For the screen, each plate was normalized to its own interquartile mean. Wells with OD_600_ of ≤3 standard deviations below their plate’s interquartile mean were considered hits.

### Generation of COL-CfrA strain

The *cfrA* coding sequence from the *E. coli* antibiotic resistance platform was codon-optimized for *Staphylococcus aureus* and placed downstream of the SarA P1 promoter [promoter sequence sourced from Boles and Horswill (31)] and optimized translation initiation region from the NTML toolkit (32). The designed insert was synthesized as a gBlock gene fragment flanked by 5′-EcoRI and 3′-BamHI restriction sites. Both insert and pKK30 vector were digested with Fastdigest EcoRI and BamHI (Thermo Fisher) per manufacturer’s instructions. Insert and pKK30 were ligated with T4 ligase (Thermo Fisher) per manufacturer’s instructions and transformed into top 10 *E. coli* on 10-µg/mL trimethoprim selection. The sequence-validated construct was then electroporated into *S. aureus* strain RN4220 and transduced into *S. aureus* strain COL using φ85 bacteriophage.

### Generation of *E. coli* Δ*bamB* Δ*tolC rlmN*::kan triple mutant

Primers AJS48/AJS49 were used to amplify the disrupted *rlmN* locus from the Keio collection mutant (33). The amplicon was then recombined into the *rlmN* locus of our BW25113 *ΔbamBΔtolC* double mutant using lambda RED-recombineering technology (34). *rlmN* disruption was validated by PCR and Sanger sequencing. The completed strain possesses an *rlmN* locus disrupted by a kanamycin resistance cassette flanked by FRT sites.

### Isolation of MTN and MTL from WAC01849

WAC01849 was inoculated into each of three baffled flasks containing 50 mL of SAM media. Cultures were incubated for 1 week at 30°C, 250 rpm. Grown cultures were diluted 1:50 into 6 L of Bennett’s media and incubated for another week at 30°C, 250 rpm, after which the conditioned media were harvested and the cell pellet was discarded. Ethyl acetate was mixed 1:1 with conditioned media and mixed well for 20 minutes; the organic layer was harvested, and the process was repeated once with fresh ethyl acetate. The aqueous layer and precipitate were discarded, and the organic layer was dried by rotary evaporation and resuspended in 11-mL DMSO.

The extract was then fractionated into 12-mL fractions by reverse-phase liquid chromatography using a 13-g FlashPure C18 column (BÜCHI Labortechnik) on a CombiFlash system (Teledyne Technologies, Inc.) with a flow rate of 30 mL/minute. The mobile phase was water + 0.1% (vol/vol) formic acid (solvent A) and acetonitrile + 0.1% (vol/vol) formic acid (solvent B). Solvent B was held constant at 10% for 3 minutes, then increased over 14 minutes to 100% solvent B. Solvent B was held at 100% for 5 minutes, then decreased over 2 minutes to 10% solvent B and held at 10% for 3 minutes. Fractions were dried by Genevac centrifugal evaporator (SP Scientific) and resuspended in DMSO. Fractions 8, 9, and 19 were found to be active.

Fraction 19 was further fractionated on a reverse-phase C18 semiprep high performance liquid chromatography (HPLC) column (XSelect CSH C18, 5-µm pore size, 10 × 100 mm column) with a flow rate of 3 mL/minute. Temperature was held constant at 30°C. The mobile phase was water + 0.1% formic acid (solvent A) and acetonitrile + 0.1% formic acid (solvent B). Solvent B was held constant at 43% for 5 minutes, then increased over 5 minutes to 95%; this was held constant for 3 minutes, then decreased over 1 minute back to 43% and held constant for 5 minutes. Three-milliliter fractions were collected starting at 5 minutes. Active fractions (fractions 7, 8, and 9) were dried separately by lyophilization. Fraction 7 yielded MTL as a cinnamon-brown color.

To purify MTN, CombiFlash fractions 8 and 9 were fractionated on a reverse-phase C18 semiprep HPLC column (XSelect CSH C18, 5 µm pore size, 10 × 100 mm column) with a flow rate of 3 mL/minute. Temperature was held constant at 30°C. The mobile phase was water + 0.1% formic acid (solvent A) and acetonitrile + 0.1% formic acid (solvent B). Solvent B was held constant at 25% for 5 minutes, then increased over 14 minutes to 32.2%; this was further increased over 1 minute to 95%, held constant for 3 minutes, then decreased over 1 minute back to 25% and held for 4 minutes. Three-millilter fractions were collected starting at 5 minutes and continued until 20 minutes. Active fractions (4 and 5) were combined, dried by lyophilization, and resuspended in methanol.

The resuspended sample was fractionated on a gravity LH-20 size-exclusion chromatography (3.66 × 43.5 cm). The mobile phase was room-temperature methanol with a 3.2-mL/min flow rate. Two hundred twenty-five milliliters of solvent was allowed to elute before collection of 36 4-mL fractions. Active fractions were combined, dried on a Genevac centrifugal evaporator (SP Scientific), and resuspended in acetonitrile.

The resuspended sample was finally fractionated on a reverse-phase C8 semiprep HPLC column (Eclipse XDB-C8, 5-µm pore size, 9.4 × 250 mm column) with a flow rate of 3 mL/minute. Temperature was held constant at 30°C. The mobile phase was water + 0.1% formic acid (solvent A) and acetonitrile + 0.1% formic acid (solvent B). Solvent B was held constant at 25% for 5 minutes, then increased over 14 minutes to 32.2%; this was further increased over 1 minute to 95%, where it was held constant for 5 minutes, then finally decreased over 1 minute to 25% and held for 4 minutes. Three-milliter fractions were collected starting at 5 minutes and continued until 20 minutes. Active fractions (9, 10, and 11) were dried separately by lyophilization. Fractions 10 and 11 yielded MTN as a wispy yellow solid.

### MTN and MTL structure elucidation

One milligram of each compound was dissolved in deuterated chloroform. Proton NMR experiments were performed on a Bruker AVIII 700 MHz equipped with a cryoprobe. Proton shifts for 8-*O*-methyltetrangomycin and 8-*O*-methyltetrangulol are summarized below and are consistent with those reported by Park *et al* (17).

**MTN**: ^1^H NMR (CDCl_3_, 500 MHz) δ 8.29 (1H, d, J = 8.0 Hz), 7.76 (1H, dd, J = 7.5, 1.0 Hz), 7.71 (1H, dd, J = 8.5, 7.5 Hz), 7.52 (1H, d, J = 8.0 Hz), 7.30 (1H, dd, J = 8.5, 1.0 Hz), 4.03 (3H, s), 3.17 (2H, s), 3.10 (1H, d, J = 15.0 Hz), 3.00 (1H, d, J = 15.0 Hz), 1.50 (3H, s).

**MTL**: ^1^H NMR (CDCl_3_, 500 MHz) δ 11.05–11.23 (1H, br. s), 8.29 (1H, d, J = 8.5 Hz), 8.11 (1H, d, J = 8.5 Hz), 7.94 (1H, dd, J = 7.5, 1.0 Hz), 7.72 (1H, dd, J = 8.5, 7.5 Hz), 7.35 (1H, d, J = 8.5 Hz), 7.24 (1H, s), 7.12 (1H, d, J = 1.0 Hz), 4.07 (3H, s), 2.49 (3H, s).

### Synergy grid assays

All synergy grid assays were performed in CAMH broth (Beckton Dickson and Company). All compounds and antibiotics were diluted to working stocks of 4× final concentration in CAMH. MTL or MTN were serial-diluted 1:2 down rows A–G of a u-bottomed 96-well plate (Sarstedt, Inc.); antibiotics were serial-diluted 1:2 across rows 11–2 in a separate 96-well plate. Twenty-five microliters from each plate was combined into a third plate to make a synergy grid at 2× concentration. Bacterial colonies were resuspended to an OD_600_ of 0.08–0.1, diluted 1:100 in CAMH, and 50 µL was added to each well to yield a final 1× concentration (with no more than 1% DMSO in any wells where necessary). Column 12 was reserved for alternating growth/sterility control wells. Completed plates were incubated at 37°C, 250 rpm for 18–20 hours after which OD_600_ was measured on a Synergy H1 microplate reader (BioTek Instruments). All synergy grid assays were performed in biological triplicates.

### MALDI experiments

The methylation state of A2503 in the presence and absence of MTN or MTL and Cfr was determined in *E. coli* BW25113 Δ*bamB*Δ*tolC* and BW25113 Δ*bamB*Δ*tolC rlmN::kan* strains following the methodology previously described (19). Briefly, BW25113 Δ*bamB*Δ*tolC* and BW25113 Δ*bamB*Δ*tolC rlmN::kan* were transformed independently with an empty pZA or a pZA vector containing the wild-type *cfrA* gene from *S. aureus* regulated under the Ptet promoter and operator. Transformed *E. coli* BW25113 Δ*bamB*Δ*tolC* cells were plated in LB agar supplemented with 100-µg/mL ampicillin, and *E. coli* BW25113 Δ*bamB*Δ*tolC rlmN::kan* cells in LB agar supplemented with 100-µg/mL ampicillin and 50-µg/mL kanamycin. A single colony was selected from each plate, and two 10-mL overnight cultures with corresponding antibiotics were grown for 18 hours. The next day, 10 mL of fresh LB with antibiotics, 30-ng/mL anhydrotetracycline hydrochloride, and 0.1% DMSO in the presence and absence of 3-µM MTL or 30-µM MTN were inoculated with 100 µL of the overnight culture, in total 10 combinations of strains, vectors, and adjuvants were made, with two to five biological repetitions per condition. Cultures were grown at 37°C for 3–4 hours until OD_600 nm_ reached 0.6–0.9, then cells were chilled on ice and centrifuged at 4°C for 10 minutes to remove media. Pelleted cells were flash-frozen, and total RNA was extracted using the total RNeasy Midi Kit (Qiagen-75144) following the vendor’s instructions. To isolate the rRNA fragments from the total RNA, 200 pmol of total RNA was annealed to 2 µmol of a DNA oligo corresponding to the complementary sequence for the *E. coli* 23S rRNA C2480-C2520 fragment in a buffer containing 75-mM HEPES, pH 7.0, 150-mM KCl. Annealed hybrids were digested in 1× mung bean nuclease buffer using 60 U of mung bean nuclease (NEB M0250L) and 5 µg of RNAse A for 1 hour at 37°C, and products were precipitated overnight at −20°C. RNA pellets from digestions were suspended in 12 µL of a 1:2 water: formamide solution; RNA loading dye (NEB B0363S) was added; samples were boiled to 90°C for 5 minutes, spun down, and loaded on 1× Tris/Borate/EDTA (TBE), 13% polyacrylamide, 8-M urea mini gels. Gels were stained for 5 minutes with 1× SybrGold (Invitrogen S11494) solution prepared in UltraPure DEPC-treated water (Invitrogen 750023) and visualized on a BioRad ChemiDoc MP. The expected rRNA fragment was sliced; the gel piece was shredded and suspended on 300 µL of 1× Tris/EDTA (TE) buffer for extraction. Extracted RNA was removed from the gel using Spin X-centrifuge tubes (Costar 8160) and precipitated overnight in the presence of 1 µL of glycoblue (Invitrogen AM9515). RNA fragments were recovered after centrifugation, resuspended in 12 µL of water and 6 µL of 0.5-M 3-hydroxypicolinic acid (3-HPA) (Thermo Scientific, 241050050), mixed with 500 U of T1 RNAse (Thermo Scientific, 01221951), and incubated at 37°C for 3 hours. After T1 digestion, HCl was added to 0.12 M, and the mix was incubated at 25°C for 30 minutes. Finally, fragments were lyophilized and resuspended in 5 µL of UltraPure water; 2 µL of this suspension was sandwiched between 2 µL of freshly made 3-HPA matrix while plating onto an MS 2-mm 384-well sample plate (Shimadzu, TO-454R00) and dried in a desiccator. Data were acquired in a Shimadzu AXIMA Performance instrument under positive ion reflectron tuning mode and analyzed using the Shimadzu MALDI-MS software. Three to five technical repetitions were collected for each biological repetition.

### *In vitro* enzyme assays

For activity assays, 4Fe4S reconstituted Cfr was produced with minor modifications of previously published protocols (19, 20, 35) using pET28b:*cfrA* and pDB128-2 vectors co-transformed in Rosetta 2(DE3)plysS grown in LB media. The 2447–2624 region of *E. coli* 23S rRNA transcript was produced, purified, and folded following reference (8). For activity, 100-µL reactions proceeded under anaerobic conditions in 100-mM HEPES, pH 8.0, 100-mM KCl, 10-mM MgCl_2_, 2-mM DTT, 20-µM flavodoxin, 2-µM flavodoxin reductase, 1.4-nCi/µL (240 mM) ^14^C SAM, 2-µM reconstituted Cfr, 2-µM 23S rRNA 2447–2625 fragment, and 1% DMSO, with or without 300-µM MTN, and 5-mM NADPH was used to initiate the reaction; no NADPH was added for a zero time point. Reactions proceeded for 90 minutes at 37°C and were quenched with 5-µL 1-M sulfuric acid. Methylated RNA was purified from the mix using Zymo RNA clean and concentrator-25 (Cat No. R1018), eluted twice in 50 µL of RNAase-free water to a final volume of 100 µL, diluted with 5 mL of Ultima Gold scintillation liquid, mixed and stored overnight in the dark before counting in a Beckman Coulter LS6500 scintillation counter. The total activity of Cfr was calculated after the subtraction of the zero time point from the Cfr and Cfr-MTN reactions.

## SUPPLEMENTAL MATERIAL

Supplemental material mbio.01791-23-s0001.docx Supplemental tables and figures.

ASM does not own the copyrights to Supplemental Material that may be linked to, or accessed through, an article. The authors have granted ASM a non-exclusive, world-wide license to publish the Supplemental Material files. Please contact the corresponding author directly for reuse.
