# Supplementary material for: Angucyclinones rescue PhLOPSA antibiotic activity by inhibiting Cfr-dependent antibiotic resistance
Source: mBio. 2023 Nov 28;14(6):e01791-23. doi: 10.1128/mbio.01791-23 (PMC10746278; doi:10.1128/mbio.01791-23)
Supplement: Supplemental material — Supplemental tables and figures. [file mbio.01791-23-s0001.docx]

**Supplementary Table S1: MIC Tables for *E. coli* BW15223 Δ*bamB*Δ*tolC***

**
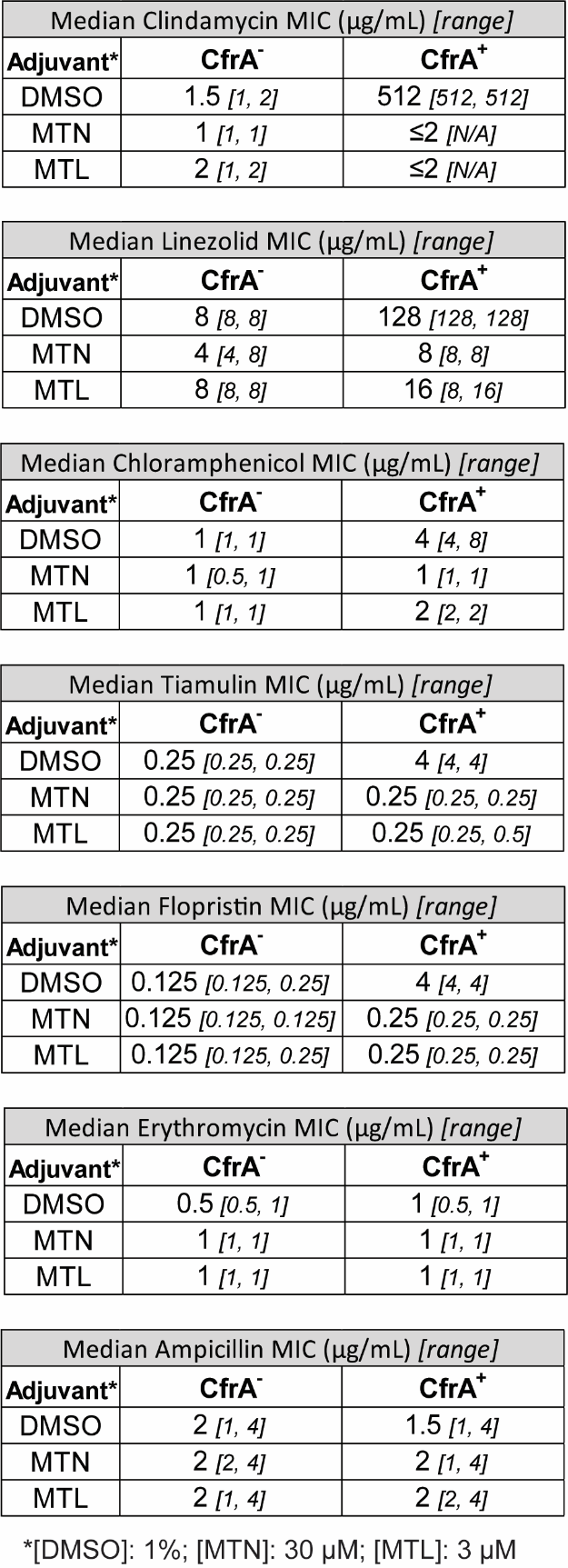
**

**Supplementary Table S2: MIC Tables for *S. aureus* COL**

**
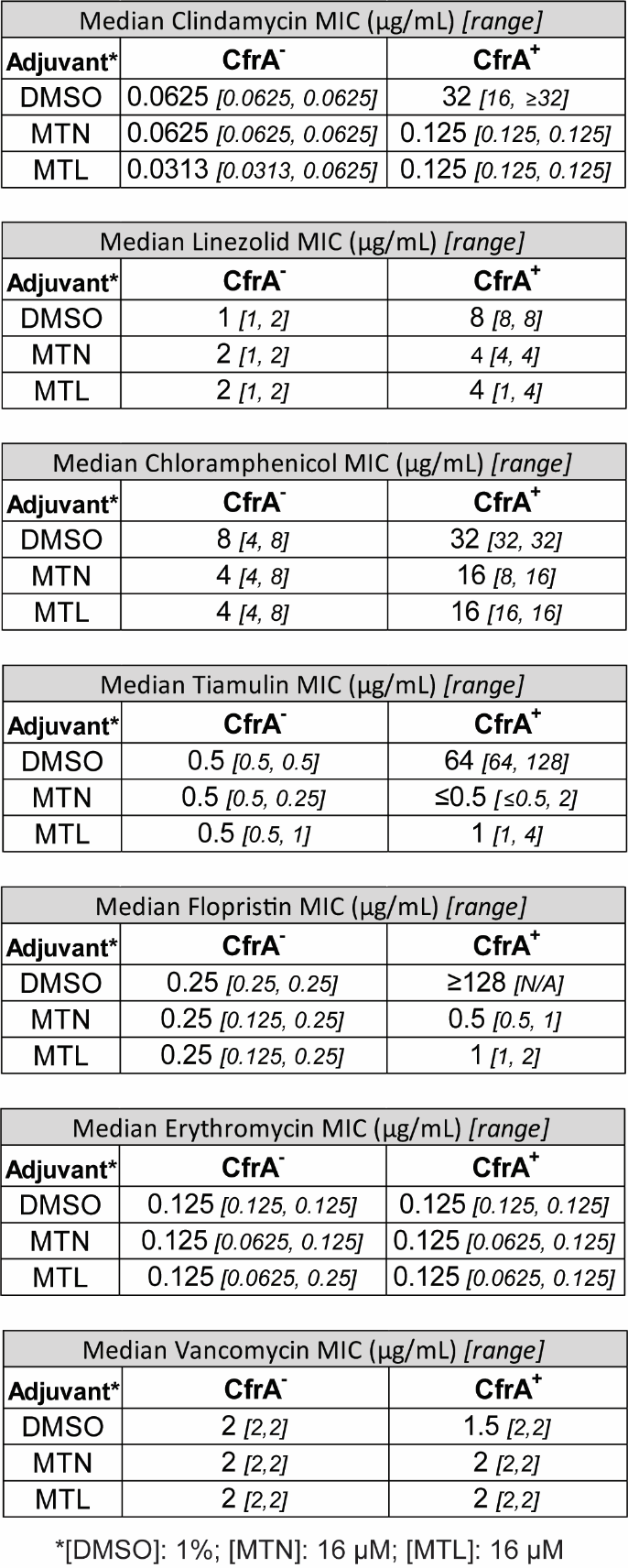
**

**Supplementary Table S3: Strains and plasmids used in this study**

| **Strain** | **Description** | **Reference** |
| --- | --- | --- |
| *E. coli* Top10 | General cloning and plasmid maintenance | Invitrogen |
| *E. coli* BW25113 Δ*bamB*Δ*tolC* | Outer membrane-weakened strain | (1) |
| *E. coli* BW25113 *rlmN::kan* | KAN^R^ cassette inserted into *rlmN* locus | (2) |
| *E. coli* BW25113 Δ*bamB*Δ*tolC* *rlmN::kan* | Outer membrane-weakened strain; KAN^R^ cassette inserted into *rlmN* locus | This work |
| *E. coli* BW25113/pKD46 | λ-RED PCR targeting host strain | (3) |
| *S. aureus* RN4220 | Intermediate strain for *S. aureus* cloning | (4) |
| *S. aureus* COL | Methicillin-resistant *S. aureus* clinical isolate | (5) |
| **Plasmids** |  |  |
| pGDP2 | ARP multicopy plasmid; MCS driven by pLAC promoter; KAN^R^ | (1) |
| pGDP2:*cfrA* | ARP multicopy plasmid; *cfrA* gene driven by pLAC promoter; KAN^R^ | (1) |
| pKK30 | *E. coli-S. aureus* shuttle vector; TMP^R^ | (6) |
| pKK30:*cfrA* | *E. coli-S. aureus* shuttle vector encoding *cfrA*; TMP^R^ | This work |
| pZA | Low copy *E. coli* expression vector Ptet, AMP^R^ | (7) |
| pZA:*cfrA* | Low copy *E. coli* expression vector Ptet, AMP^R^ encoding *S. aureus cfrA* | (8) |
| pET28b:*cfrA* | Medium copy *E. coli* expression vector, KAN^R^; For expression of His-SUMO-Cfr, IPTG inducible | (8) |
| pDB128-2 | *E. coli* Expression vector for FeS biogenesis containing iscS, iscU, iscA, hscA, hscB, and fdx; ARA inducible | (9) |

**Supplementary Table S4: gBlock and primers used in this study**

| **Name** | **Sequence (5’-3’)** | **Description** |
| --- | --- | --- |
| SarAp1-TIR-CfrA | gctatt**GAATTC**CTGATATTTTTGACTAAACCAAATGCTAACCCAGAAATACAATCACTGTGTCTAATGAATAATTTGTTTTATAAACACTTTTTTGtttACTTCTCATTTTTAATTAGTTATAATTAACTAAATAATAGAGCATTAAATATATTTAATAAAACTTATTTAATGCAAAATTATGACTAACATATCTATAATAAATAAAGATTAGATATCAATATATTATCGGGCAAATGTATCGAGCAAGATGCATCGGGAACAAAAGCTCGTATGATTAACTTTATAAGGAGGAAAAACATATGAATTTCAATAACAAGACGAAGTACGGAAAGATCCAGGAGTTTTTACGTTCTAACAATGAACCTGATTACAGAATCAAACAGATAACTAATGCAATCTTTAAACAGCGTATCTCTCGTTTCGAAGACATGAAAGTATTGCCTAAGTTGTTACGAGAAGATTTAATAAATAATTTTGGTGAAACAGTTTTGAATATAAAGTTGTTAGCTGAACAGAACTCTGAGCAAGTCACAAAAGTATTGTTCGAGGTGAGTAAGAACGAGCGTGTGGAGACGGTAAACATGAAATACAAGGCTGGATGGGAAAGTTTCTGCATCTCTTCTCAGTGCGGATGTAACTTTGGCTGTAAATTCTGCGCGACGGGTGATATAGGATTAAAAAAAAACTTAACAGTTGATGAGATCACTGATCAAGTTTTGTATTTTCATTTATTGGGCCATCAAATTGACTCAATTTCATTCATGGGTATGGGAGAGGCATTGGCTAACCGTCAGGTCTTCGACGCATTGGACTCATTTACGGACCCGAATTTATTCGCATTGTCTCCACGTAGATTATCTATCTCTACGATCGGCATAATCCCAAGTATTAAGAAAATCACGCAAGAATATCCTCAGGTCAATTTAACATTCAGTTTACATTCACCGTATTCTGAGGAGCGATCAAAATTAATGCCTATCAACGACCGTTACCCAATCGACGAGGTGATGAACATATTGGATGAGCACATACGATTAACATCTAGAAAAGTATATATTGCGTATATCATGTTACCAGGTGTCAATGATTCTTTGGAGCACGCGAACGAGGTTGTATCATTATTAAAAAGTAGATATAAGAGTGGAAAATTATACCATGTTAACTTGATCCGTTATAACCCCACGATCAGTGCGCCGGAAATGTATGGTGAGGCAAATGAGGGACAGGTTGAAGCTTTCTATAAGGTCTTAAAGTCAGCAGGCATACATGTCACTATCCGATCTCAATTTGGAATAGACATCGATGCGGCGTGTGGTCAGTTGTATGGCAATTACCAGAACAGTCAGTTAGAACATCATCATCATCATCATTAG**GGATCC**gaaggt | gBlock cassette for constitutive expression of *cfrA* in *S. aureus*  SarA p1 promoter  Translation Initiation Region (TIR)  *cfrA* (*S. aureus* codon-optimized, 6x HIS-tagged) |
| Primers |  |  |
| pKK30-MCS-F | GGTGTTCATCAATCGAAAAAGCAACGTAT | pKK30 multi-cloning site diagnostic primer |
| pKK30-MCS-R | ATACATACACAAGCAAAAATAGCGGTGATTG | pKK30 multi-cloning site diagnostic primer |
| RlmN-Keio-F | CAACCCTCCAGGGGCCATAAC | RlmN knockout insert for λ RED |
| RlmN-Keio-R | TACATAAAGTAACCGTGGCGTAATGGC | RlmN knockout insert for λ RED |
| RlmN-F | TGCCCGCGCACCCGTTAATAATTT | RlmN locus diagnostic primer |
| RlmN-R | CCGTCTGTGTGCTCGTGACAAAG | RlmN locus diagnostic primer |
| C2480-C2520 | GATGTGATGAGCCGACATCGAGGTGCCAAACACCGCCGTCG | Complementary sequence for rRNA fragment |


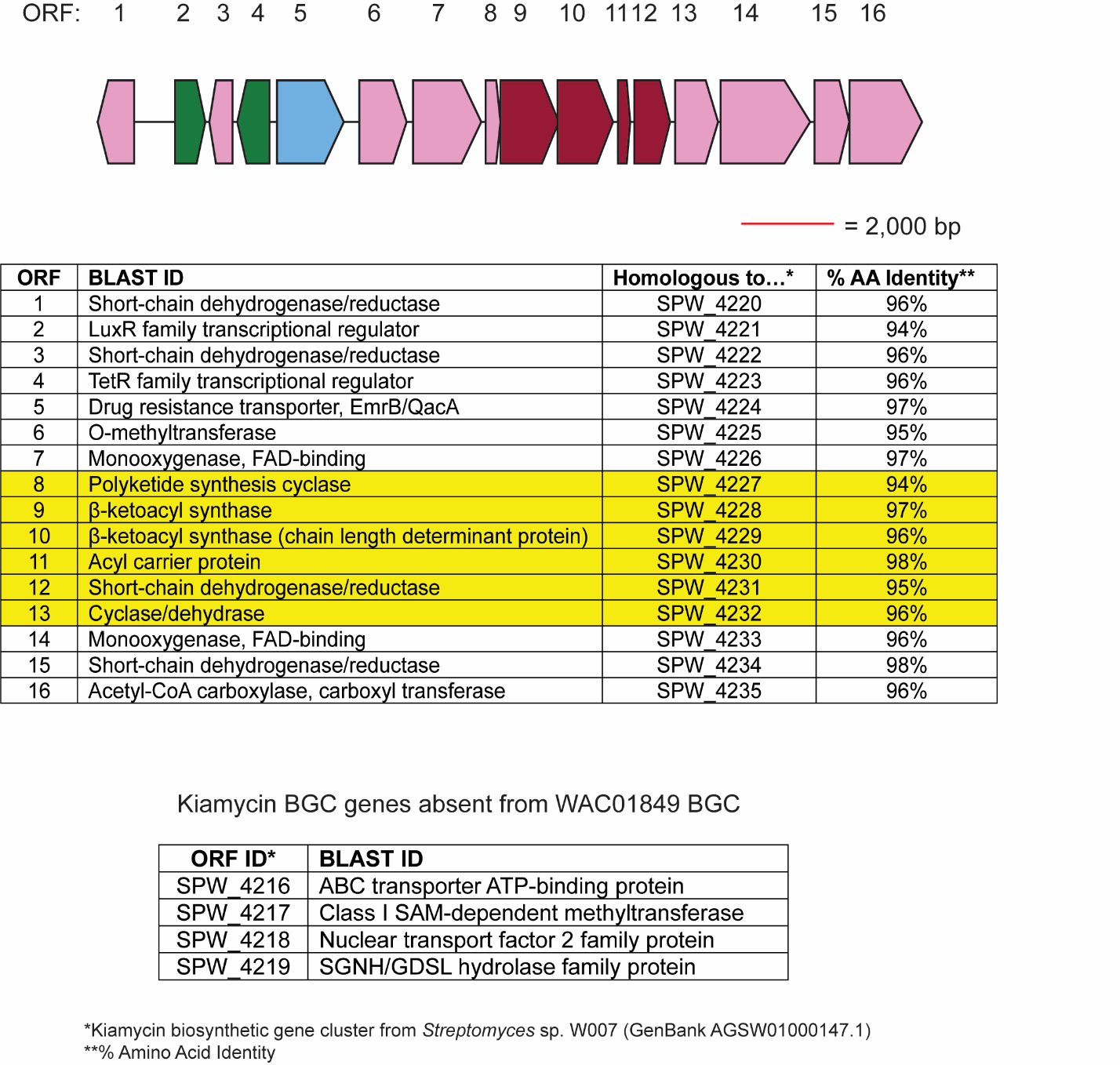


**Supplemental Figure S1: putative MTN and MTL biosynthetic gene cluster.** Genes to create the benz[a]anthraquinone core are highlighted in yellow.


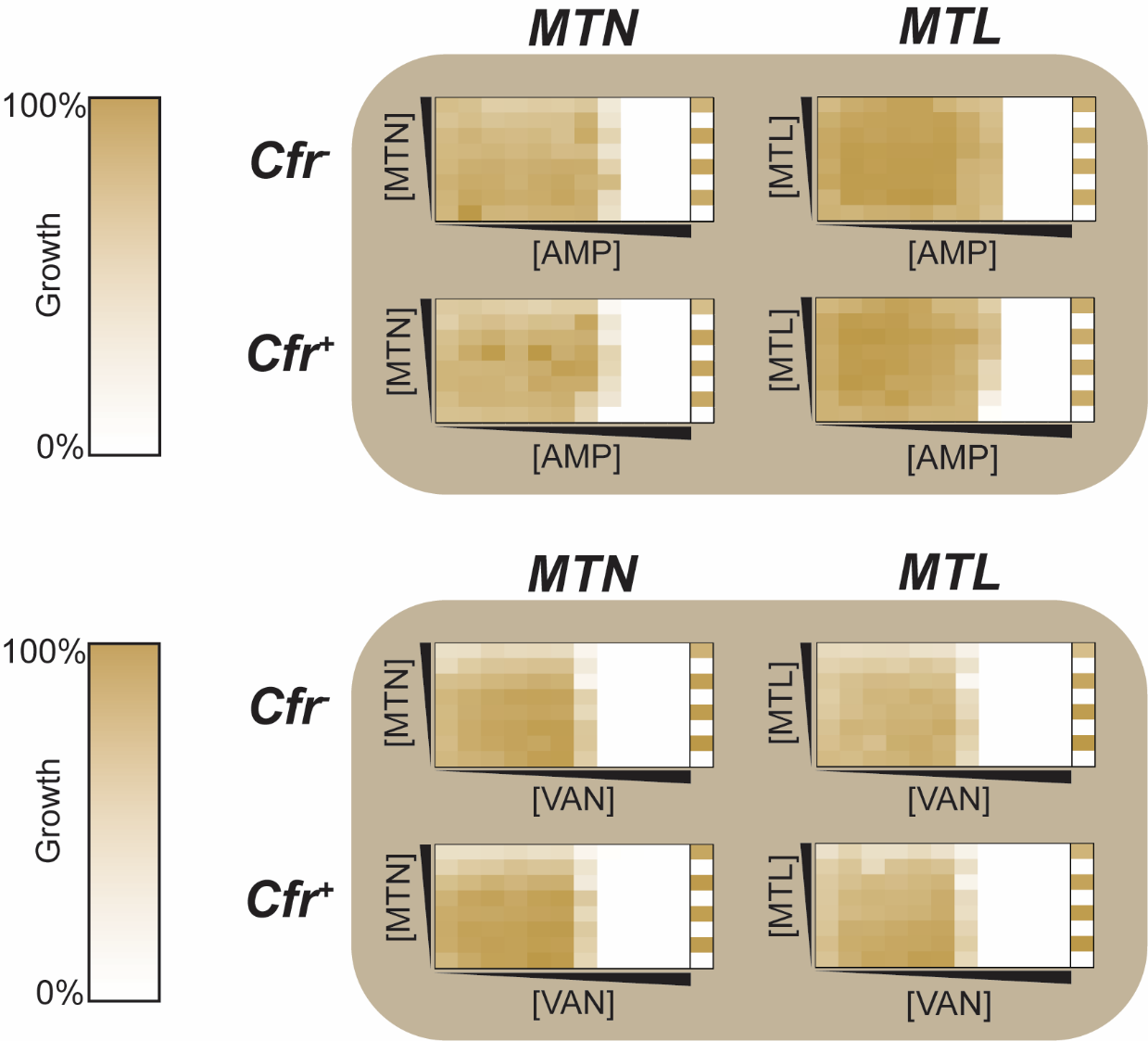


**Supplemental Figure S2: MTN and MTL fail to potentiate cell wall-acting antibiotics.** (*Top*) Synergy grids of *E. coli* Δ*bamB*Δ*tolC* +/- pGDP2:*cfr* against ampicillin. Max [AMP] = 8 µg/mL, max [MTN] = 30 µM, and max [MTL] = 3 µM. (*Bottom*) Synergy grids of *S. aureus* COL +/- pKK30:*cfr* against vancomycin. Max [VAN] = 16 µg/mL, max [MTN] = 16 µM, and max [MTL] = 16 µM. All synergy grids are representative of 3 biological replicates.

***
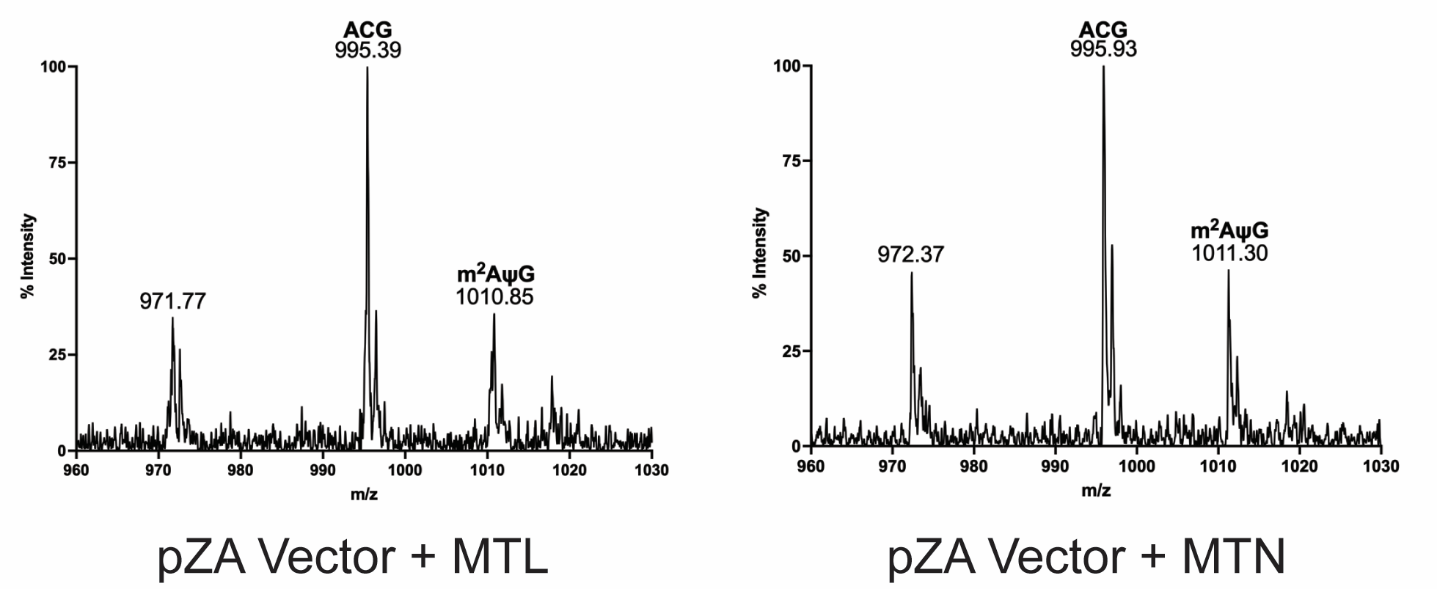
***

**Supplemental Figure S3: MTL and MTN fail to inhibit RlmN-dependent methylation.** *E. coli* BW25113 Δ*bamB*Δ*tolC* carrying the empty pZA vector treated with 3 µM MTL (*left*) or 30 µM MTN (*right*). A2503 from the 23S rRNA denoted by AψG specie.

***
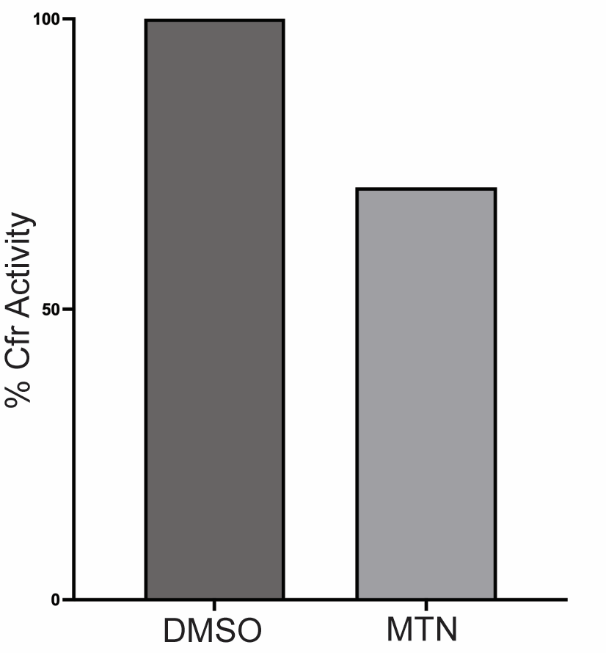
***

**Supplemental Figure S4: MTN displays modest inhibition of Cfr *in vitro*.** Purified Cfr enzyme was incubated with *E. coli* 23S rRNA transcript (region 2447-2624) and ^14^C SAM in the presence of DMSO or 300 µM MTN.

***References***

1. Cox G, Sieron A, King AM, De Pascale G, Pawlowski AC, Koteva K, Wright GD. 2017. A Common Platform for Antibiotic Dereplication and Adjuvant Discovery. Cell Chem Biol 24:98–109.

2. Baba T, Ara T, Hasegawa M, Takai Y, Okumura Y, Baba M, Datsenko KA, Tomita M, Wanner BL, Mori H. 2006. Construction of *Escherichia coli* K‐12 in‐frame, single‐gene knockout mutants: the Keio collection. Mol Syst Biol 2.

3. Datsenko KA, Wanner BL. 2000. One-step inactivation of chromosomal genes in *Escherichia coli* K-12 using PCR products. Proceedings of the National Academy of Sciences 97:6640–6645.

4. Kreiswirth BN, Löfdahl S, Betley MJ, O’Reilly M, Schlievert PM, Bergdoll MS, Novick RP. 1983. The toxic shock syndrome exotoxin structural gene is not detectably transmitted by a prophage. Nature 305:709–712.

5. Gill SR, Fouts DE, Archer GL, Mongodin EF, DeBoy RT, Ravel J, Paulsen IT, Kolonay JF, Brinkac L, Beanan M, Dodson RJ, Daugherty SC, Madupu R, Angiuoli S V., Durkin AS, Haft DH, Vamathevan J, Khouri H, Utterback T, Lee C, Dimitrov G, Jiang L, Qin H, Weidman J, Tran K, Kang K, Hance IR, Nelson KE, Fraser CM. 2005. Insights on Evolution of Virulence and Resistance from the Complete Genome Analysis of an Early Methicillin-Resistant *Staphylococcus aureus* Strain and a Biofilm-Producing Methicillin-Resistant *Staphylococcus epidermidis* Strain. J Bacteriol 187:2426–2438.

6. Krute CN, Krausz KL, Markiewicz MA, Joyner JA, Pokhrel S, Hall PR, Bose JL. 2016. Generation of a Stable Plasmid for *In Vitro* and *In Vivo* Studies of Staphylococcus Species. Appl Environ Microbiol 82:6859–6869.

7. Stojković V, Noda-Garcia L, Tawfik DS, Fujimori DG. 2016. Antibiotic resistance evolved via inactivation of a ribosomal RNA methylating enzyme. Nucleic Acids Res 44:8897–8907.

8. Tsai K, Stojković V, Noda-Garcia L, Young ID, Myasnikov AG, Kleinman J, Palla A, Floor SN, Frost A, Fraser JS, Tawfik DS, Fujimori DG. 2022. Directed evolution of the rRNA methylating enzyme Cfr reveals molecular basis of antibiotic resistance. Elife 11.

9. Stojković V, Fujimori DG. 2015. Radical SAM-Mediated Methylation of Ribosomal RNA, p. 355–376. *In* .
